# Supplementary material for: Details Matter: Noise and Model Structure Set the Relationship between Cell Size and Cell Cycle Timing
Source: Front Cell Dev Biol. 2017 Nov 3;5:92. doi: 10.3389/fcell.2017.00092 (PMC5675860; doi:10.3389/fcell.2017.00092)
Supplement: Supplementary file 1 [file DataSheet1.pdf]

Supplementary Material:

Details Matter: Noise and model structure set the relationship between cell size and cell cycle timing

Felix Barber [1], **Po-Yi Ho** [2], **Andrew W. Murray** [1,3,*] **and Ariel Amir** [2,*]

*Correspondence:
Ariel Amir, School of Engineering and Applied Sciences, Harvard University, 29 Oxford Street, Cambridge, 02138 MA, USA
arielamir@seas.harvard.edu

Andrew W. Murray: awm@mcb.harvard.edu

1 METHODS

1.1 Simulations

Simulations were implemented using a discretized time format, with normally distributed noises included where noted in the main text. To mimic experimental conditions, we seeded populations with 500 cells that were randomly selected from the final populations of separate, discretized generation based simulations. These seeding generation based simulations were each run for 9 generations. We simulated growth and proliferation of these seeded populations for roughly 6 volume doubling times. To ensure convergence of the discretized generation seeding populations we compared statistics from the final population for simulations in which cell volume was allowed to decrease with analytic calculations of the V_b vs. V_d slopes. These calculations were not included in this text due to their deviation from the simulations presented herein, that enforced monotonically increasing cell volume. Once appropriate simulation lengths were established, these were then applied to the simulations in which cell volume monotonically increased to generate the results presented in the main text. The total number of cells used to generate final statistics per individual simulation was approximately 3.5×10^4. Unless otherwise stated, simulations were repeated a minimum of 50 times per condition to generate the reported statistics.

In all simulations of a noisy asymmetry growth model for budding cells, we assumed a constant volume growth rate, with $\sigma_\lambda \to 0$. In simulations of either noisy asymmetry or noisy timing growth models for budding cells, the volume growth rate for each cell was fixed over that cell's entire cell cycle. All stochastic variables simulated throughout the text were assumed to be independent and identically distributed for each cell cycle, with the exception of the initiator accumulation model in bacteria, in which the two daughter cells from a given division event had the same stochastically generated noise ξ_i in initiation of DNA replication. In all simulations we set the concentrations $c_1 = c_2 = 1$, since as mentioned in the main text these parameters are simply involved in setting the scale for the cell size distributions. Unless explicitly stated otherwise, all budding simulation results presented herein imposed the condition that cell volume monotonically increased.

2 DERIVATIONS

Here we derive the relation $\langle I_b^X \rangle = \langle \tilde{\Delta} \rangle$ that was used in the main text to derive equation 8:

$$\begin{aligned}\langle I_b^{X,n+1} \rangle &= \frac{1}{2} \times \left(\langle I_b^{M,n+1} \rangle + \langle I_b^{D,n+1} \rangle \right) \\ &= \frac{1}{2} \times \langle I_b^{M,n+1} + I_b^{D,n+1} \rangle \\ &= \frac{1}{2} \times \langle I_b^{X,n} + \tilde{\Delta} \rangle \\ \Rightarrow \langle I_b^X \rangle &= \langle \tilde{\Delta} \rangle\end{aligned} \tag{S1}$$

Here the third line holds since for any given generation of cells there is a one to one mapping in the population between mothers and daughters. Further, the sum of inhibitor abundance at birth for any given mother-daughter pair is simply the inhibitor at birth for the cell in the previous generation plus the inhibitor produced during that previous cell cycle. Assuming stationarity of the stochastic process, with $\langle I_b^{X,n+1} \rangle = \langle I_b^{X,n} \rangle$, we obtain the desired result.

We now derive the result that for $r = 1$, $\langle V_i^D \rangle = 2\langle A_c \rangle / c_2$, assuming no noise in r for simplicity. This result is used in Section 3.3.2 of the main text.

$$\begin{aligned}\langle V_i^{X,n+1} \rangle &= \frac{1}{2} \times \left(\langle V_i^{M,n+1} \rangle + \langle V_i^{D,n+1} \rangle \right) \\ &= \frac{1}{2} \times \left((1+r)\langle V_i^{X,n} \rangle - r\langle V_i^{X,n} \rangle + 2\frac{\langle A_c \rangle}{c_2} \right) \\ \Rightarrow \langle V_i^X \rangle &= \frac{2\langle A_c \rangle}{c_2} \\ \langle V_i^D \rangle &= \langle V_b^D \rangle + \frac{\langle A_c \rangle - \langle A_b^D \rangle}{c_2} \\ &= \frac{\langle A_c \rangle}{c_2} + \langle V_i^X \rangle \frac{r}{1+r} \\ r = 1 \Rightarrow \langle V_i^D \rangle &= 2\frac{\langle A_c \rangle}{c_2}\end{aligned} \tag{S2}$$

Here the second line comes from applying equations 2 and 3 in the main text. The third line comes from assuming stationarity of the stochastic process. The 5th line comes from applying equation 3 from the main text, combined with the observation that $\langle A_b^D \rangle = r^2 \langle V_i^X \rangle / (1 + r)$. Taking $r = 1$ gives the desired result.

3 SUPPLEMENTARY TABLES AND FIGURES

3.1 Figures

As mentioned in the main text, there are a variety of different choices to be made when modeling an inhibitor dilution process in budding yeast. In the main text we assumed noisy integrator for inhibitor synthesis, with noisy asymmetry in cell growth post Start. However, we could have instead adopted a noisy synthesis rate model for synthesis and a noisy timing model for cell growth. Of the model variants tested, the model presented in the main text with uncorrelated noise in r and inhibitor production $\tilde{\Delta}$ was

most effective in maintaining robust adder slopes of approximately 1 for the biologically relevant range of parameter values.

Figure S1 compares the changes in the predicted V_b vs. V_d slopes with variation in model parameters for a selection of different models. This is done by displaying the maximum and minimum changes in the predicted V_b vs. V_d slope when a single specified model parameter (called y here for generality) is varied over the biologically relevant parameter range for that variable $[y_{min}, y_{max}]$. Note that y_{min} and y_{max} values for all model parameters are listed below. Here a change in slope for a given parameter set (i.e. for a single point in parameter space with $y = y'$) is calculated relative to the slope predicted for that same parameter set, subject to the substitution $y = y_{min}$. We defined maximal and minimal changes in slope as the largest positive and negative changes in the predicted V_b vs V_d slope over the range $y_{min} \leq y' \leq y_{max}$. We note that these maximal and minimal changes were always calculated varying only y, with *all other variables fixed.* A box and whisker plot for each model variable y is generated from the distribution of these maximum and minimum relative changes, where each datapoint used to generate the plot comes from performing the above max-min calculation for a different position in parameter space. Together these positions comprise a uniformly spaced array of points spanning the biologically relevant parameter space for each model, so that the distribution of these min and max values for the complete dataset provides a rough insight of the degree of sensitivity of the V_b vs. V_d slope to variation in the parameter being probed. This provides a means of contrasting three distinct inhibitor dilution models. To generate the data for this figure, noise strengths were sampled from the following intervals, consistent with data in Figure S2: $\sigma_\lambda/\langle\lambda\rangle \in (0.0, 0.3)$, $\sigma_t/t_{db} \in (0.0, 0.16)$, $\sigma_x/\langle x\rangle \in (0.0, 0.3)$, $\sigma_s/\langle\tilde{\Delta}\rangle \in (0.0, 0.3)$, $\sigma_K/\langle K\rangle \in (0.0, 0.3)$, $\sigma_\Delta/\langle\tilde{\Delta}\rangle \in (0.0, 0.3)$. Budded growth parameters were sampled from $\langle x\rangle \in (0.5, 0.7)$ and $\langle t\rangle \in (0.585, 0.765)$ to give the same sampled range of asymmetry ratios r for all models in the noiseless limit. Each interval was discretized by 6 points per parameter. Slopes inferred for each given parameter set were averaged from 3 repeats.

From Figure S1 (A) we see that a noisy synthesis rate model with a noisy timing growth model is robust to noise in passage through Start $\sigma_s/\langle\tilde{\Delta}\rangle$ and is insensitive to noise in synthesis rate $\sigma_K/\langle K\rangle$, but displays substantial increases in the observed slope for non-zero noises in budded phase duration (σ_t/t_{db} and growth rate $\sigma_\lambda/\langle\lambda\rangle$). We also observe further increases in slope with increasing lengths of the budded phase. From (B) we observe that a noisy integrator synthesis model with a noisy timing growth model is robust to noise in passage through Start and is insensitive to noise in inhibitor synthesis, but displays significant increases in observed slope for increasing budded phase duration, noise in budded phase duration, and noise in growth rate. Finally from (C) we observe that a noisy integrator model with a noisy asymmetry growth model is robust to noise in passage through Start and insensitive to noise in inhibitor synthesis $\sigma_\Delta/\langle\tilde{\Delta}\rangle$. We also observe variation with $\langle x\rangle$ and $\sigma_x/\langle x\rangle$. Of these variants, model (C) shows a weaker dependence on variation in cell division asymmetry $\langle x\rangle$ than (A) and (B) do for their equivalents: $\langle t\rangle/t_{db}$, σ_t/t_{db} and $\sigma_\lambda/\langle\lambda\rangle$. This difference may be studied in greater depth by comparing plots of the variation in V_b vs. V_d slope for the different models discussed here, and leads us to favor model (C) as the most consistent with adder behavior in budding yeast.

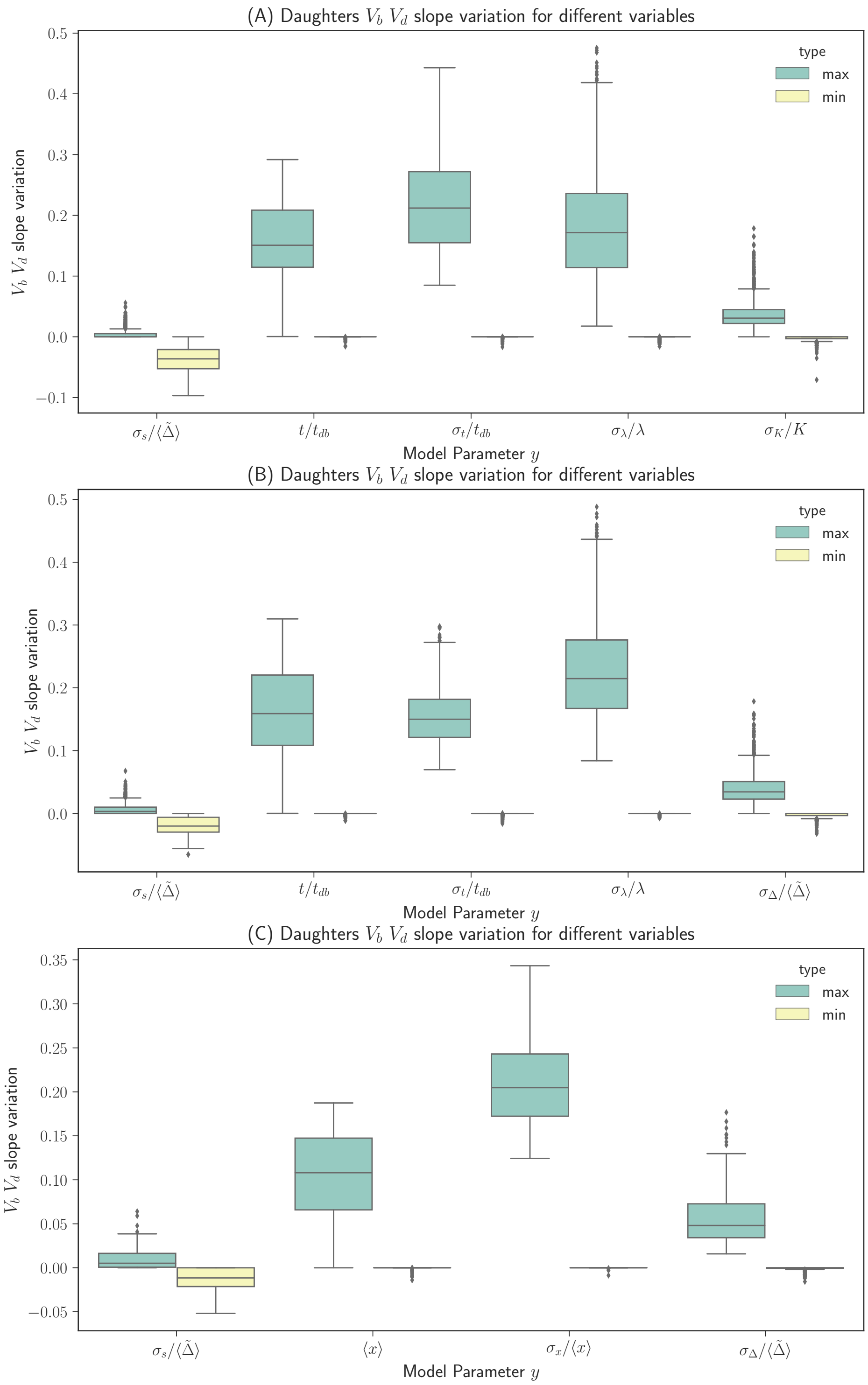

Figure S1. An inhibitor dilution model with uncorrelated noise in $\langle x\rangle$ and $\tilde{\Delta}$ is the most robust of the model variants considered in asymmetrically dividing budding cells. Box and whisker plots display the sensitivity to variation in model parameters for selected inhibitor dilution models in asymmetrically dividing, budding cells. All plots are for daughter cells only. Dots represent outliers. (A) A noisy synthesis rate model with noisy timing for growth. (B) A noisy integrator model for inhibitor synthesis, with noisy timing growth. Both (A) and (B) are sensitive to noise in growth rate $\sigma_\lambda/\langle\lambda\rangle$, budded time σ_t/t_{db} and to variation in the average budded length $\langle t\rangle/t_{db}$. (C) A noisy integrator model for inhibitor synthesis, with noisy asymmetry for growth. This displays some dependence on the cell division asymmetry $\langle x\rangle$ and $\sigma_x/\langle x\rangle$. All models were insensitive to noise in inhibitor production $\sigma_\Delta/\langle\tilde{\Delta}\rangle$ (or $\sigma_K/\langle K\rangle$ for model (A)) and were robust to noise in passage through Start $\sigma_s/\langle\tilde{\Delta}\rangle$.

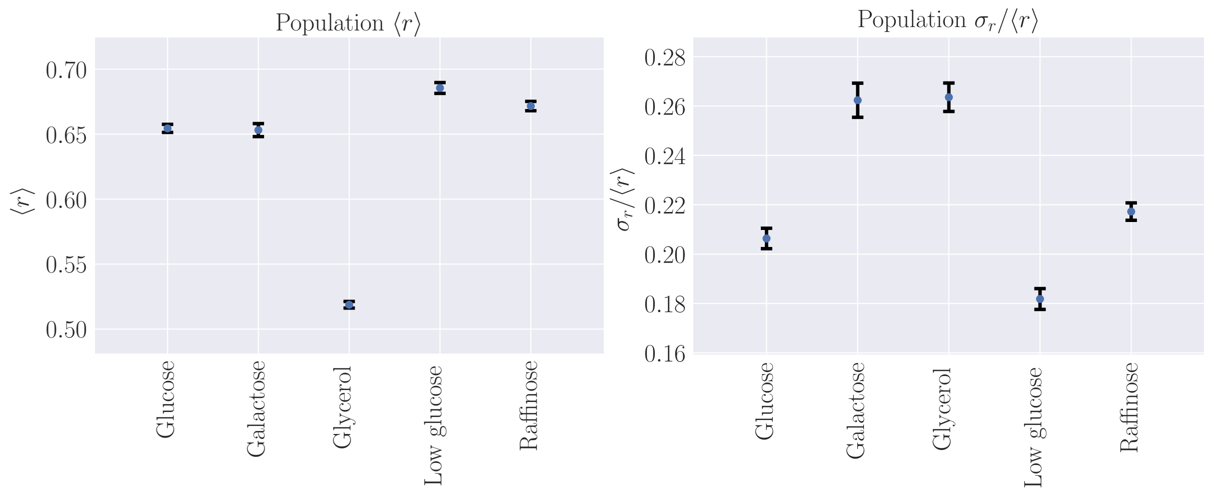

Figure S2. Observations of relevant model parameters based on measurements of diploid cells across a range of growth media. (A) Mean asymmetry ratio r. (B) Coefficient of variation for the asymmetry ratio $\sigma_r/\langle r\rangle$. (C) Coefficient of variation for the single cell volume growth rate $\sigma_\lambda/\langle\lambda\rangle$. (C) Standard deviation of the duration of the budded portion of the cell cycle σ_t/t_{db}, normalized by the cell volume doubling time $t_{db} \equiv \langle\lambda\rangle/\ln(2)$. Error bars are standard error. Data was obtained from Ilya Soifer, and was previously published in Soifer et al. (2016).

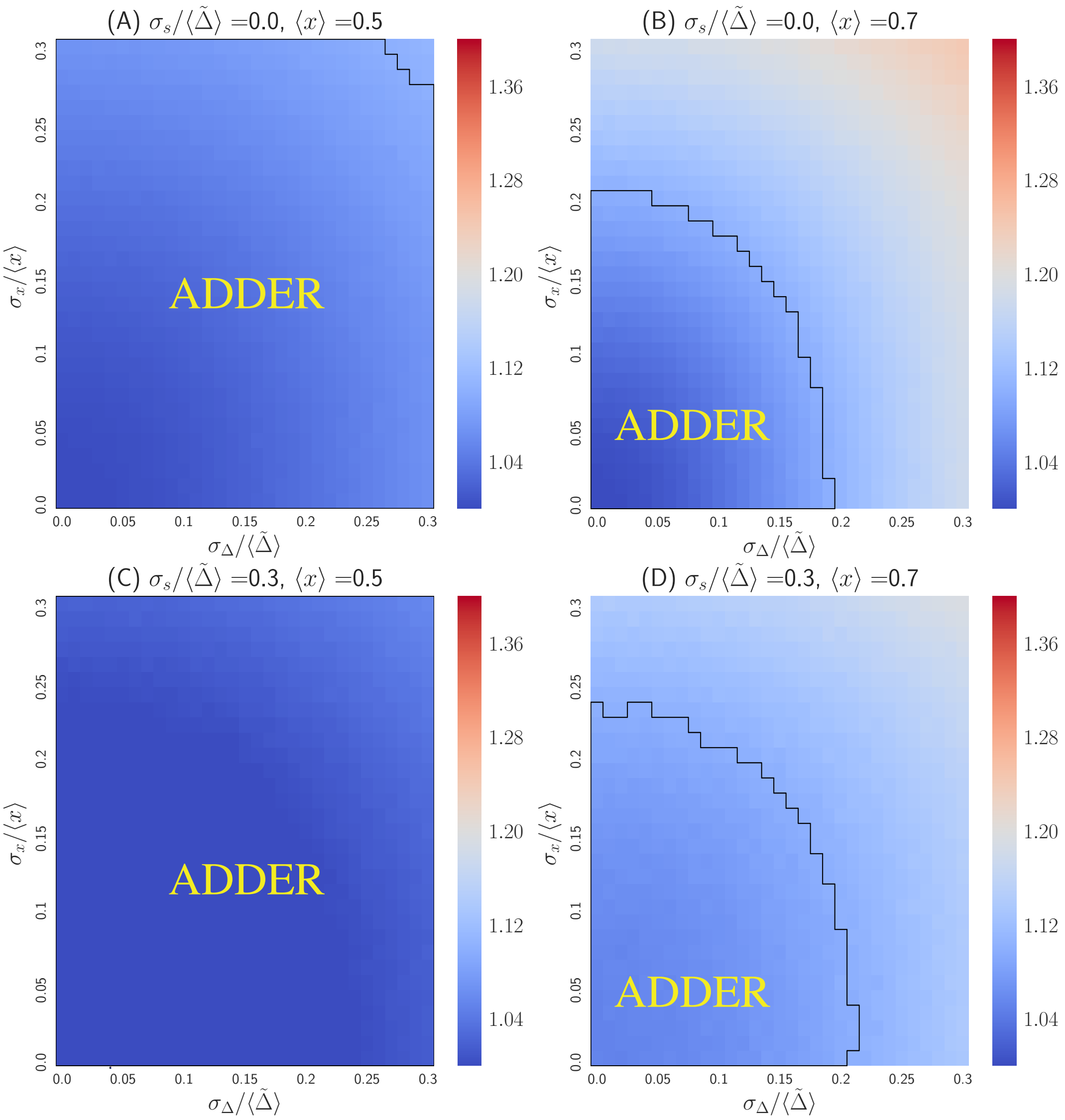

Figure S3. The inhibitor dilution model yields robust adder behavior for asymmetrically dividing, budding mother cells. Heat maps of linear regression slopes from fitting V_b vs. V_d for mother cells from populations simulated using an inhibitor dilution model. Inhibitor synthesis is modeled using the noisy integrator model, and we assume noisy asymmetry for growth in the budded portion of the cell cycle, as outlined in the main text. Variation is with respect to $\sigma_\Delta/\langle\tilde{\Delta}\rangle$ and $\sigma_x/\langle x\rangle$. $\sigma_s/\langle\tilde{\Delta}\rangle$ and $\langle x\rangle$ are as labeled. Black outlines provide a guide to the eye for regions in which adder-like behavior is observed (slope $= 1.0 \pm 0.1$).

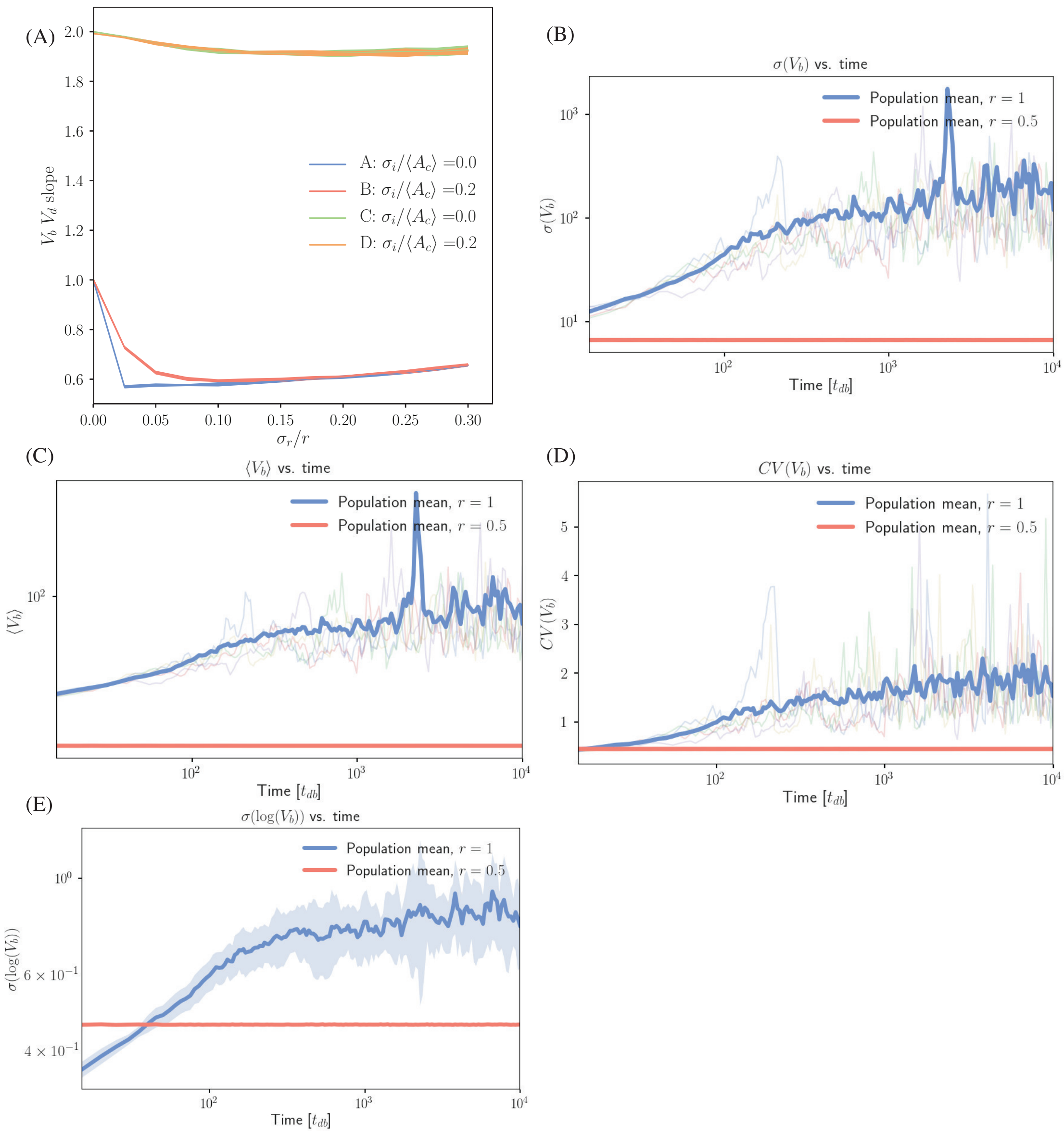

Figure S4. An initiator accumulation model in symmetrically dividing, budding cells yields poor size regulation. (A) Plots of full population linear regression slopes of V_b vs. V_d in a symmetrically dividing initiator accumulation model with $r = 1$ for variable σ_r/r and $\sigma_i/\langle A_c\rangle$. Lines A and B were obtained allowing cell volume to decrease before Start. Lines C and D are obtained by forcing cell growth to increase monotonically. Linear regression slopes are ≤ 2 for finite noise strengths, indicating very weak size control for symmetrically dividing, budding cells. (B-E) Population level statistics tracked for symmetrically and asymmetrically dividing, budding cells growing with an initiator accumulation model. Sequential growth and dilution steps allowed the population to be grown for 2.5×10^5 doubling times. (B) $\sigma(V_b)$ vs. time (in t_{db}) shows an increase in $\sigma(V_b)$ by approximately two orders of magnitude in contrast to the asymmetrically dividing control, with the mean traces for the 20 replicate simulated populations shown for both symmetric ($\langle x\rangle = 1$) and asymmetric ($\langle x\rangle = 0.5$) growth. Faded lines show 5 randomly selected single traces. (C) The same for $\langle V_b\rangle$ vs. time (in t_{db}). (D) The same for $CV(V_b)$ vs. time (in t_{db}). (E) $\sigma(\log(V_b))$ vs. time (in t_{db}) shows that the increase in standard deviation is below that expected from a pure geometric random walk in volume, with the mean for 20 repeats shown in bold. Noise was introduced in x, passage through Start and inhibitor production with $\sigma_x/\langle x\rangle = 0.2$, $\sigma_i/\langle A_c\rangle = 0.0$. Shaded error represents standard deviation of the 20 repeats tracked per condition. The saturation of the increase in average volume and standard deviation are inconsistent with a geometric random walk, but demonstrate substantial spreading of the cell size distributions. This is consistent with very weak size control.

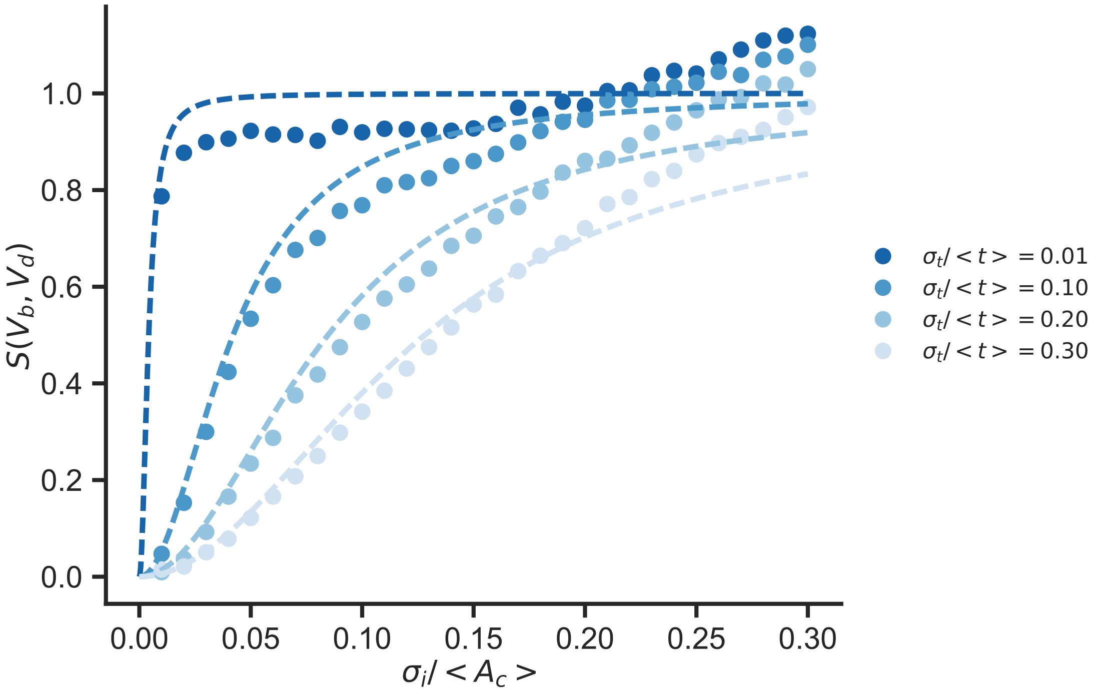

Figure S5. Comparison of the approximate expression (Eq. 13 in the Main Text) and numerical results for the linear regression slopes of V_b versus V_d in the initiator accumulation model for symmetrically dividing bacterial cells. Circles plot numerical results. Dashed lines plot Eq. 13 in the Main Text. Colors denote $\sigma_t/\langle t\rangle$ as indicated in the legend.
